# Genetic analysis of a Yayoi individual from the Doigahama site provides insights into the origins of immigrants to the Japanese Archipelago

**DOI:** 10.1038/s10038-024-01295-w

**Published:** 2024-10-15

**Authors:** Jonghyun Kim, Fuzuki Mizuno, Takayuki Matsushita, Masami Matsushita, Saki Aoto, Koji Ishiya, Mami Kamio, Izumi Naka, Michiko Hayashi, Kunihiko Kurosaki, Shintaroh Ueda, Jun Ohashi

**Affiliations:** 1 Department of Biological Sciences, Graduate School of Science, The University of Tokyo, Tokyo, 113-0033 Japan; 2 Department of Legal Medicine, Toho University School of Medicine, Tokyo, 143-8540 Japan; 3 The Doigahama Site Anthropological Museum, Yamaguchi, 759-6121 Japan; 4 Medical Genome Center, National Research Institute for Child Health and Development, Tokyo, 157-8535 Japan

**Keywords:** Population genetics, Genetic variation

## Abstract

Mainland Japanese have been recognized as having dual ancestry, originating from indigenous Jomon people and immigrants from continental East Eurasia. Although migration from the continent to the Japanese Archipelago continued from the Yayoi to the Kofun period, our understanding of these immigrants, particularly their origins, remains insufficient due to the lack of high-quality genome samples from the Yayoi period, complicating predictions about the admixture process. To address this, we sequenced the whole nuclear genome of a Yayoi individual from the Doigahama site in Yamaguchi prefecture, Japan. A comprehensive population genetic analysis of the Doigahama Yayoi individual, along with ancient and modern populations in East Asia and Northeastern Eurasia, revealed that the Doigahama Yayoi individual, similar to Kofun individuals and modern Mainland Japanese, had three distinct genetic ancestries: Jomon-related, East Asian-related, and Northeastern Siberian-related. Among non-Japanese populations, the Korean population, possessing both East Asian-related and Northeastern Siberian-related ancestries, exhibited the highest degree of genetic similarity to the Doigahama Yayoi individual. The analysis of admixture modeling for Yayoi individuals, Kofun individuals, and modern Japanese respectively supported a two-way admixture model assuming Jomon-related and Korean-related ancestries. These results suggest that between the Yayoi and Kofun periods, the majority of immigrants to the Japanese Archipelago originated primarily from the Korean Peninsula.

## Introduction

The prehistory of the Japanese Archipelago is represented by the “Jomon period”, a Neolithic period. The name “Jomon”, meaning “rope pattern”, reflects the distinctive feature of the Jomon culture, characterized by pottery with a unique pattern created using rope [1]. Although there are various opinions about the duration of the Jomon period, archeological evidence widely supports that it started approximately 16,500 years ago and persisted in isolation from the Eurasian continent for at least 10,000 years [2]. The main subsistence activities during the Jomon period were hunting and gathering. Rice cultivation in paddy fields was introduced to northern Kyushu, Japan, at the end of the Final Jomon period, about 3000 years ago, marking the beginning of the Yayoi period. It then gradually spread throughout Japan from the Middle to Late Yayoi period [3].

There were various hypotheses to explain the history of the Japanese. For example, the “transformation model” posits that only culture, not people, came from the continent. The “replacement model” suggests a complete replacement of indigenous Jomon people by the Yayoi people, while the “hybridization model” proposes admixture between indigenous Jomon people and continental immigrants [4]. Currently, the “dual-structure model,” one of the hybridization models proposed by Hanihara [5] based on the skeletal characteristics of ancient Jomon and Yayoi individuals, is widely accepted.

Strong evidence supporting the dual-structure model has been accumulated through population genetic studies. The presence of not only the mitochondrial DNA (mtDNA) and Y chromosome haplogroups commonly found in continental East Asians, but also the mtDNA haplogroups such as N9b and M7a [6–8] and Y chromosome haplogroups such as D-M125 [9–11], which are uncommon in continental Eurasians but common in Jomon people, supports the hypothesis of hybridization between Jomon people and continental Eurasian immigrants in the Japanese Archipelago. Studies on single nucleotide polymorphisms (SNPs) on autosomes have suggested that the genetic component of modern Japanese can be explained as a mixture of Ainu-related ancestry and continental East Asian-related ancestry [12, 13], providing additional evidence for the dual-structure model. Furthermore, studies revealing a closer genetic affinity between the Ainu of Hokkaido Prefecture and the Ryukyuans of Okinawa Prefecture despite their geographical distance [14–16] suggest the migration of continental Eurasians to the mainland of Japan. The dual-structure model is considered fundamentally correct; however, among Mainland Japanese, there exists genetic heterogeneity [17–20] partly due to variations in the proportion of Jomon-related ancestry [21]. This indicates that the admixture of Jomon and continental Eurasian immigrants did not uniformly progress throughout the mainland of Japan.

Several studies have also investigated the origins of the Jomon people through the analysis of ancient nuclear genomes. The analysis of Jomon genomes revealed that the Jomon lineage is located basal to the other East Eurasian lineages [22–24]. However, the origins of continental Eurasian immigrants who migrated to the Japanese Archipelago from the Yayoi to the Kofun period remain unclear due to the lack of high-quality nuclear genome data from Yayoi individuals.

A recent study [25] reported a sudden change in the genetic profile of the Japanese between the Yayoi and Kofun periods, using newly reported genomes of three Kofun individuals and previously published genomes of two Yayoi individuals from the Shimomotoyama rock shelter in Japan [26]. Based on the differences in genetic components between the Yayoi and Kofun individuals, the authors suggested that gene flow occurred from Northeastern Siberians during the Yayoi period, and independent gene flow from East Asians, such as Han Chinese, occurred during the Kofun period. Consequently, they proposed a three-way admixture model for modern Mainland Japanese.

However, certain aspects of the analysis presented in the paper proposing a three-way admixture model [25] require further consideration. A major concern is the representativeness of the Yayoi samples used. Human skeletal remains from the Yayoi period, excavated from northern Kyushu and its surrounding areas, have been categorized into two main groups based on metric analyses [27, 28]. The Yayoi people from northwestern Kyushu area (including Nagasaki and nearby regions) exhibit morphological traits similar to those of the Jomon people, such as a low face and short stature [29–31]. In contrast, the Yayoi people from northern Kyushu and Yamaguchi areas are characterized by a higher face and taller stature compared to the Jomon people [32–34], suggesting a significant genetic influence from immigrants. The Yayoi samples used in the previous study [25], specifically Shimomotoyama 2 and Shimomotoyama 3, belong to the northwestern Kyushu Yayoi group and have been shown to possess genetic components from both the Jomon people and immigrants from the Asian continent [26]. To gain a comprehensive understanding of the genetic characteristics of the Yayoi people, it is also essential to investigate samples from the northern Kyushu and Yamaguchi Yayoi group. Additionally, the sequence data quality for Shimomotoyama 2 and Shimomotoyama 3 was not high, with coverage below 0.1x. Consequently, the question of whether there is a significant genetic difference between Yayoi people and Kofun people remains open for further study.

In this study, we determined the whole nuclear genome sequence of an ancient Yayoi individual from the Doigahama site in Yamaguchi Prefecture, Japan. This individual belongs to the northern Kyushu and Yamaguchi Yayoi group. To clarify the origins of continental Eurasian immigrants from the Yayoi to the Kofun period, we conducted further population genetic analyses on the Doigahama Yayoi individual, including admixture modeling.

## Materials and methods

### Sample

At the Doigahama site, an early to mid-Yayoi period cemetery in Doigahama, Houhoku-cho, Shimonoseki City, Yamaguchi Prefecture (Fig. S1A), bones from over 300 Yayoi individuals have been found. This study conducted DNA analysis on specimen ST1604, sourced from the Doigahama site, which encompassed nearly the entire skeletal structure. The age of ST1604 was considered to be a young adult, as evidenced by the openness of both the inner and outer tables of the three main sutures. The sex of ST1604 was inferred as female from the absence of a ridge on the supraorbital arch and the greater angle of the greater sciatic notch. With the maximum length of the femur, the height was estimated to be around 149 cm. Notably, ST1604 displayed distinctive traits, such as a long-shaped face and tall stature, diverging from the typical morphological characteristics of the Jomon people.

The ^14^C age of the ST1604 sample was 2305±20years BP (YU-13168). The calibrated ages (95.4% probability) were in the range of 405–361 cal BC (90.7%), 275–263 cal BC (3.1%), and 243–235 cal BC (1.7%). In this study, the ^14^C dates were converted into calibrated ages using the program OxCal v4.4 [35] and the international calibration datasets of IntCal20 [36].

In this paper, the ST1604 sample will henceforth be referred to as the D1604 sample, the subject of DNA analysis in this study. This study was approved by the Ethics Committee of Toho University School of Medicine (A23103_A20110_A18099_A18056).

### DNA extraction, NGS library construction, and sequencing

DNA was extracted from the right petrous bone of D1604, and the next-generation sequencing (NGS) library was constructed as described in our previous study [37]. While performing DNA extraction, purification, and NGS library construction, all possible precautions were taken to avoid contamination. Experiments were performed in a laboratory that was exclusively dedicated to ancient DNA research and physically isolated from other molecular research laboratories. All the experiments were performed in a laminar flow cabinet that was routinely irradiated with UV light. Frequent surface cleaning was performed routinely before and after the experiments. We sampled the dense part of petrous bones around the cochlea by first removing the outer part using the sanding machine (Dremel, USA), grinding the clean inner part into fine powder with a mill (Multi-Beads Shocker MB601U; Yasui Kikai, Japan). The powdered samples (690 mg) were decalcified in 0.5 M EDTA (pH 8.0) for 2 h at 56 °C in a rotating oven, and the supernatant was removed by centrifugation. The decalcification step was performed thrice, followed by DNA extraction. DNA was extracted using phenol: chloroform: isoamyl alcohol (25:24:1) followed by an extraction with an equal volume of chloroform. After centrifugation, the aqueous solution was removed and concentrated to a final volume of 200 μl by centrifugation dialysis using the Amicon Ultra-15 centrifugal filter (Merck Millipore). Then, we transferred the supernatant to a MiniElute silica spin column (QIAGEN, Germany).

Using the DNA obtained, we prepared single- and double-stranded NGS libraries. These libraries were sequenced on the MiSeq platform (Illumina, USA) using the MiSeq Reagent Kit v3 150 cycles. After checking ancient DNA authenticity, we sequenced the single-stranded library on an Illumina NovaSeq instrument at Macrogen Japan in the 150-bp paired-end sequencing design.

To analyze the mitogenome sequence, we performed in-solution target enrichment for the double-stranded library using the SureSelect custom kit designed for the human mitogenome (Agilent Technologies, USA) as described in previous studies [37–39]. The enriched library was sequenced on the MiSeq platform (Illumina, USA) using the MiSeq Reagent Kit v3 (150 Cycles).

### Estimation of mtDNA haplogroup

We assessed a pattern of postmortem chemical modifications expected for ancient DNA using mapDamage v2.0.6 [40], and Mitosuite v1.0.9 [41]. We also estimated the concordance of mitogenome sequence based on the definitive haplogroup sites using Mitosuite v1.0.9 [41].

### Sequence data processing of NGS data

We used the single-stranded library for the nuclear genome analysis. We trimmed the Illumina adapter sequences of the raw fastq files of the D1604 sample and filtered out reads shorter than 30 bp using AdapterRemoval v2.3.0 [42] with parameters “--trimns --trimqualities --minlength 30 --minquality 25 --minadapteroverlap 1”. Then, we mapped the adapter-clipped merged reads to the human reference genome hs37d5 using the aln and samse modules in the BWA (Burrows-Wheeler Aligner) v0.7.17 [43] with parameters “-l 16500 -n 0.01 -o 2”, the recommended parameters for ancient DNA [44]. We sorted the output BAM file and discarded reads with a Phred-scaled mapping quality score lower than 30, using SAMtools v1.18 [45]. PCR duplicates were then removed using MarkDuplicates from the Genome Analysis Toolkit v4.3.0.0 [46].

After clipping 8 bp from both ends of all reads using the trimBam function on bamUtils v1.0.15 [47], we called pseudo-haploid genotype data by randomly sampling a single high-quality base per site included in the 1240 K panel [48], using SAMtools mpileup with “-R -B -q30 -Q30” option and pileupCaller v1.5.2 (https://github.com/stschiff/sequenceTools) in “randomHaploid” mode. We merged the output file with the previously published genotype data explained in the section below, using PLINK v1.90b [49]. The pseudo-haploid genotype data of the D1604 sample are available upon request from the authors.

### Sex identification

Sex of D1604 was estimated based on the number of mapped reads to the X and Y chromosomes. The *R*_Y_ index, Y/(X + Y), of D1604, was calculated using the “idxstats” command in SAMtools v1.18 [45], and the genetic sex was determined in a manner consistent with a previous study [50].

### Dataset compilation

We downloaded the 1240 K datasets v54.1.p1 from Allen Ancient DNA Resource (AADR) [51] (https://reich.hms.harvard.edu/allen-ancient-dna-resource-aadr-downloadable-genotypes-present-day-and-ancient-dna-data). We used Human Genome Diversity Panel (HGDP) data [52] and Simons Genome Diversity Panel (SGDP) data [53] as modern population panels. We extracted these individual genotype data from the downloaded 1240 K datasets, and then removed duplicated individuals from the SGDP dataset. In addition, we randomly excluded one member from each pair with a KING kinship coefficient [54] exceeding 0.0884, using PLINK v2.00a5 [55]. We also excluded the China_Lahu outlier (HGDP01319) since the result of PCA appeared unreliable. Details about modern populations used in our analyses are provided in Table S1.

We also extracted ancient Eurasian genomes [24, 25, 56–58] from 1240 K datasets and additionally merged ancient genomes from Japan or Korea [23, 26, 59]. Details about the ancient Japanese and Korean individuals used in our analyses are provided in Table S2, and their excavation sites are mapped in Figure S1.

### Population clustering analysis

We performed a principal component analysis (PCA) using smartpca v16000 from the EIGENSOFT package v7.2.1 [60]. We projected the ancient genomes onto the PC space calculated from modern individuals using the “lsqproject: YES” option. Additionally, we applied the “shrinkmode: YES” option when the modern population set included only East Eurasian populations.

For unsupervised model-based genetic clustering, we employed the ADMIXTURE v1.3.0 program [61]. Before running ADMIXTURE, we carried out the SNP filtering process. First, we extracted modern East Asian and Siberian populations, as well as ancient Japanese and ancient Korean individuals. Subsequently, we filtered out SNPs with minor allele frequency lower than 0.01 with PLINK v1.90b flag “--maf 0.01”. Following that, we conducted pruning for linkage disequilibrium (LD) with a window size of 200 SNPs, advanced by 50 SNPs, and established an r2 threshold of 0.2, using PLINK v1.9 flag “--indep-pairwise 200 25 0.2”. This LD pruning retained 122,381 SNPs. Then, we ran ADMIXTURE v1.3.0 with the “--cv=100” option for each value of ancestry number *K*, ranging from 2 to 5.

We reconstructed a maximum likelihood phylogenetic tree using TreeMix v1.3 [62]. We combined the Jomon individuals into a unified group labeled “Jomon” to draw the simple phylogenetic tree. We included modern individuals or populations: French, Papuan, Mixe, Ulchi, Korean, Japanese, Han, She, Ami, as well as ancient individuals or populations: Doigahama_Yayoi, Japan_Honshu_Kofun, Korea_Gimhae_DaeseongDong, and Jomon. After excluding SNPs without calling information in any of the groups, a total of 940,570 SNPs remained. We ran the TreeMix program with the following options, “-bootstrap -k 500 -noss -root French”.

### *f*-statistics

To assess the genetic relationships between Doigahama Yayoi individual, D1604, and other East Eurasian populations, we computed *f*-statistics using the qp3Pop v651 program and the qpDstat v980 program in ADMIXTOOLS v7.0.2 package [63].

First, we calculated *f*3(Mbuti; Doigahama_Yayoi, X), the outgroup *f*3 with Mbuti pygmy as an outgroup, where X was one of the populations in our dataset (Tables S1 and S2). This outgroup *f*3 was used to identify the modern population with the closest genetic affinity to the Doigahama Yayoi sample. Then, we computed *f*4-statistics [64] in the form *f*4(Mbuti, Doigahama_Yayoi; modern Korean or modern Japanese, X) to rigorously test the hypothesis that no population surpasses modern Korean or modern Japanese populations in terms of genetic affinity with Doigahama Yayoi. In these analyses, *Z* value, the deviation of the *f*-statistic from zero in units of the standard error, was also calculated.

Additionally, we computed *f*4(Mbuti, Doigahama_Yayoi; Jomon1, Jomon2) to investigate the Jomon subpopulation demonstrating exceptionally high genetic affinity with the Doigahama Yayoi individual. The Jomon1 and Jomon2 subpopulations were selected from those listed in Table S2.

### Admixture modeling

**Fig. 1:**
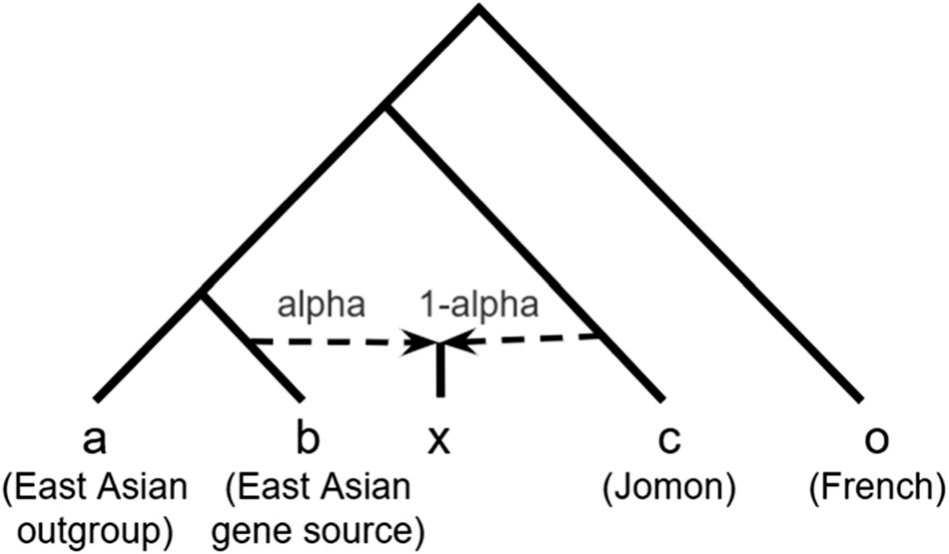
Modeling of *f*4 ratio test. Tree model of the *f*4 ratio test to determine the admixture proportions “alpha” and “1-alpha” from source populations “b” and “c”

We consolidated the data from seven Jomon populations explained in Table S2 into a unified group labeled “Jomon” for our admixture modeling analysis. We assessed the compatibility of the admixture model and estimated the admixture ratio of Doigahama Yayoi using the qpF4ratio v400 program in the ADMIXTOOLS v7.0.2 package [63]. We configured French as outgroup “o”. We tested the admixture models for both ancient and modern Japanese (“x”) with potential East Eurasian gene flow sources (“b”) and Jomon sources (“c”). Detailed explanations of the assumed model are provided in Fig. 1.

We also ran qpWave and qpAdm v1520 in the ADMIXTOOLS v7.0.2 package to find the admixture model with the most suitable sources of ancestry or gene flow from continental Eurasian populations. In this analysis, we ran the programs without using “allsnps: YES” to avoid the bias that the option may introduce [65]. We used the following populations as the outgroup for qpAdm modeling: Mbuti, Italy_North_Villabruna_HG [66], Iran_GanjDareh_N [67], Tianyuan [68], USR1 [69], Ami, Bianbian_EN, Liangdao2_EN [70], and She. We ran the qpWave program to assess the independence of outgroup populations, with a threshold of *P* = 0.01. After confirming the independence of outgroup populations, we tested a two-way admixture model, with Jomon and continental East Asian populations as sources of ancestry for ancient and modern Japanese populations using the qpAdm program. Furthermore, we assessed a previously suggested three-way admixture model, using Jomon, East Asian, and Northeast Asian populations as sources of ancestry for the target population [25]. We included the “China_HMMH_MN” individual, proposed as the potential gene flow source in the previous study [25], as the source of the Northeast Asian-related ancestry and determined the suitability of the three-way admixture model.

## Results

### Sample genome information

Before conducting population genetic analyses, we assessed the quality of the D1604 genome file to verify the reliability of the data. Tables S3 and S4 provide precise numerical indicators to assess the quality. The autosomal mean coverage of D1604 slightly exceeded 2x, with approximately 84.5% (= 971,776/1,150,639) of autosomal SNPs from 1240 K SNPs covered (Table S4). This is particularly noteworthy as the coverage of other published Yayoi genomes [26, 57] in the dataset does not exceed 0.1x and the number of covered 1240 K SNP does not surpass 50,000 SNPs.

We assessed the genetic sex of the D1604 sample based on the number of mapped reads to sex chromosomes, as previously reported [50]. In this method, a sample is classified as female if its upper bound of the 95% confidence interval (CI) of *R*_Y_ index is below 0.016, and as male if its lower bound of the 95% CI of *R*_Y_ index is above 0.075. The *R*_Y_ index of D1604 was 0.00051 (Table S3). Since the upper bound of the 95% confidence interval (CI) for *R*_Y_ was 0.00053, which is less than 0.016, the genetic sex of D1604 was estimated to be female, consistent with the morphological assessment.

### mtDNA haplogroup

We obtained a complete mitogenome sequence of D1604 with an average depth of 297×. The percentage of concordance across the mitogenome was > 0.993. We confirmed that the sequence exhibited the ancient DNA-like deamination pattern (Fig. S2). The mtDNA haplogroup of D1604 was estimated to be D4h1a2. Most sub-haplogroups of D4h were reported to be predominant in North/Central China (i.e., D4h1b, D4h1d, D4h1e, and pre-D4h3b) [71].

### Genetic similarity of the Doigahama Yayoi individual with ancient and present Korean populations

**Fig. 2:**
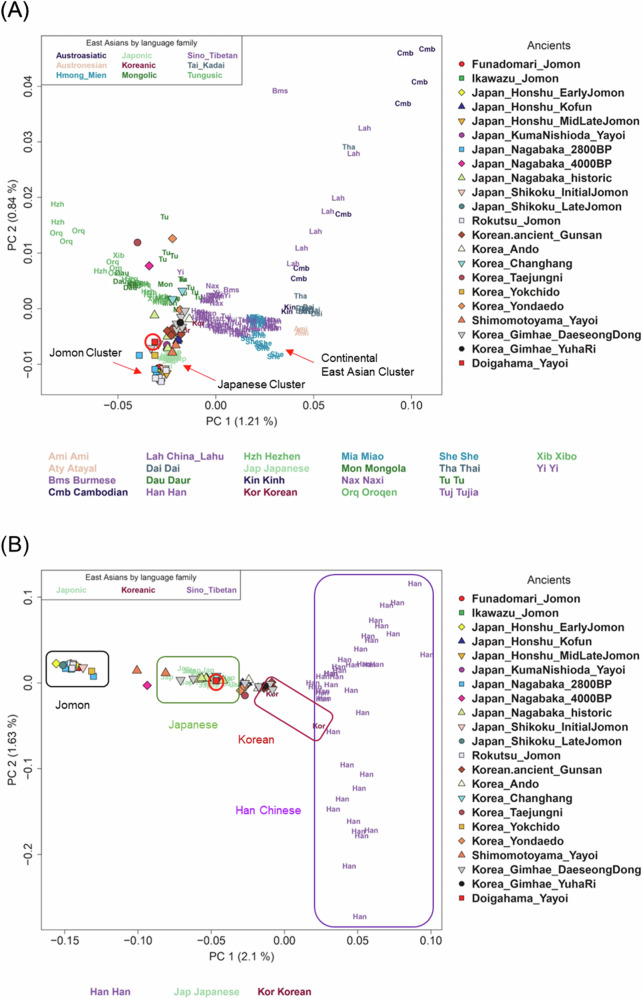
PCA plot of East Asians. The position of D1604 on the plot is indicated by a red circle. Modern East Asian populations are color-coded by language family. **A** PCA plot using all modern East Asian populations. **B** PCA plot using only Japanese, Korean, and Han Chinese as modern populations

The PCA plots, including the Doigahama Yayoi individual along with ancient and modern East Asians, are shown in Fig. 2. Jomon individuals were distinctly positioned apart from the other East Asian populations examined here (Fig. 2A), suggesting that the ancestors of Jomon people initially diverged from other East Asians. The Doigahama Yayoi individual, along with Kofun individuals, was located within the modern Japanese cluster, positioned between the Jomon and continental East Asian clusters. The PCA only for Japanese, Korean, and Han Chinese individuals indicated that the Doigahama Yayoi individual was genetically closer to both modern and ancient Korean individuals than to Han Chinese (Fig. 2B).

### Genetic ancestry of the Doigahama Yayoi individual

**Fig. 3:**
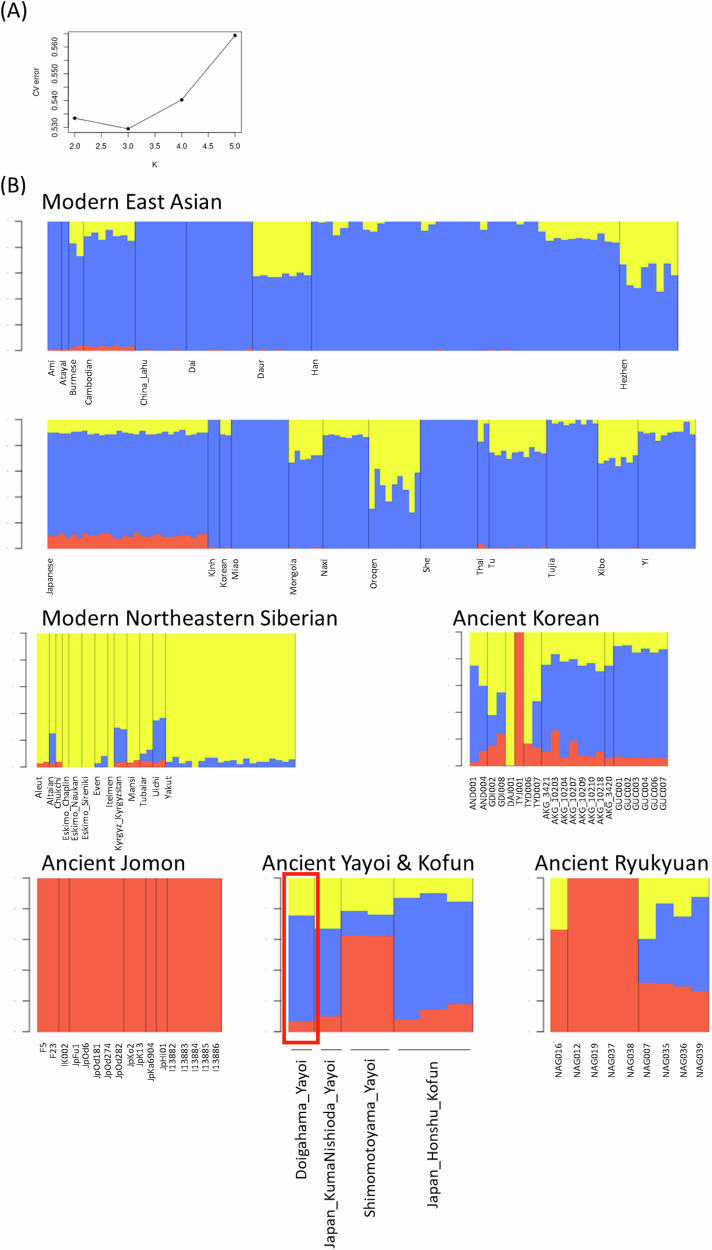
ADMIXTURE analysis using East Asian and Siberian populations. **A** Cross-validation error for ancestry number K. **B** Admixture bar plots showing estimated cluster membership values for East Asians and Northeastern Siberians at *K* = 3

In the ADMIXTURE analysis, the genetic ancestry of the Doigahama Yayoi individual was inferred together with modern East Asians, Northeastern Siberians, and ancient individuals from the Japanese Archipelago and Korean Peninsula. Based on cross-validation error values, we determined that *K* = 3 was the most plausible number of ancestral source populations for the present population set (Fig. 3A). In the admixture bar plot, the red, blue, and yellow components were interpreted as indicating components derived from Jomon-related, East Asian-related, and Northeastern Siberian-related ancestries, respectively.

As previously reported [e.g., [25, 72], modern Japanese exhibited three genetic components. Among 28 modern Japanese individuals, the average proportions of Jomon-related, East Asian-related, and Northeastern Siberian-related ancestries were approximately 10%, 80%, and 10%, respectively. The Doigahama Yayoi individual had approximately 7% Jomon-related, 67% East Asian-related, and 26% Northeastern Siberian-related ancestries. The proportions were similar to those of the Kumanishioda Yayoi individual, Kofun individuals, and modern Japanese. The observation of a significant amount of East Asian-related component in the Doigahama Yayoi individual contradicts the aforementioned previous study [25], which asserted that populations with East Asian-related ancestry migrated to the Japanese Archipelago during the Kofun period.

Interestingly, some ancient Korean individuals had considerable Jomon-related ancestry (Fig. 3B). For instance, the individual excavated from Yokchido (TYJ001) showed almost 100% of the Jomon ancestry, as reported in previous studies [57]. In contrast, modern Korean individuals exhibited little of the Jomon-related ancestry.

### Phylogenetic relationship of the Doigahama Yayoi individual with East Asian populations

**Fig. 4:**
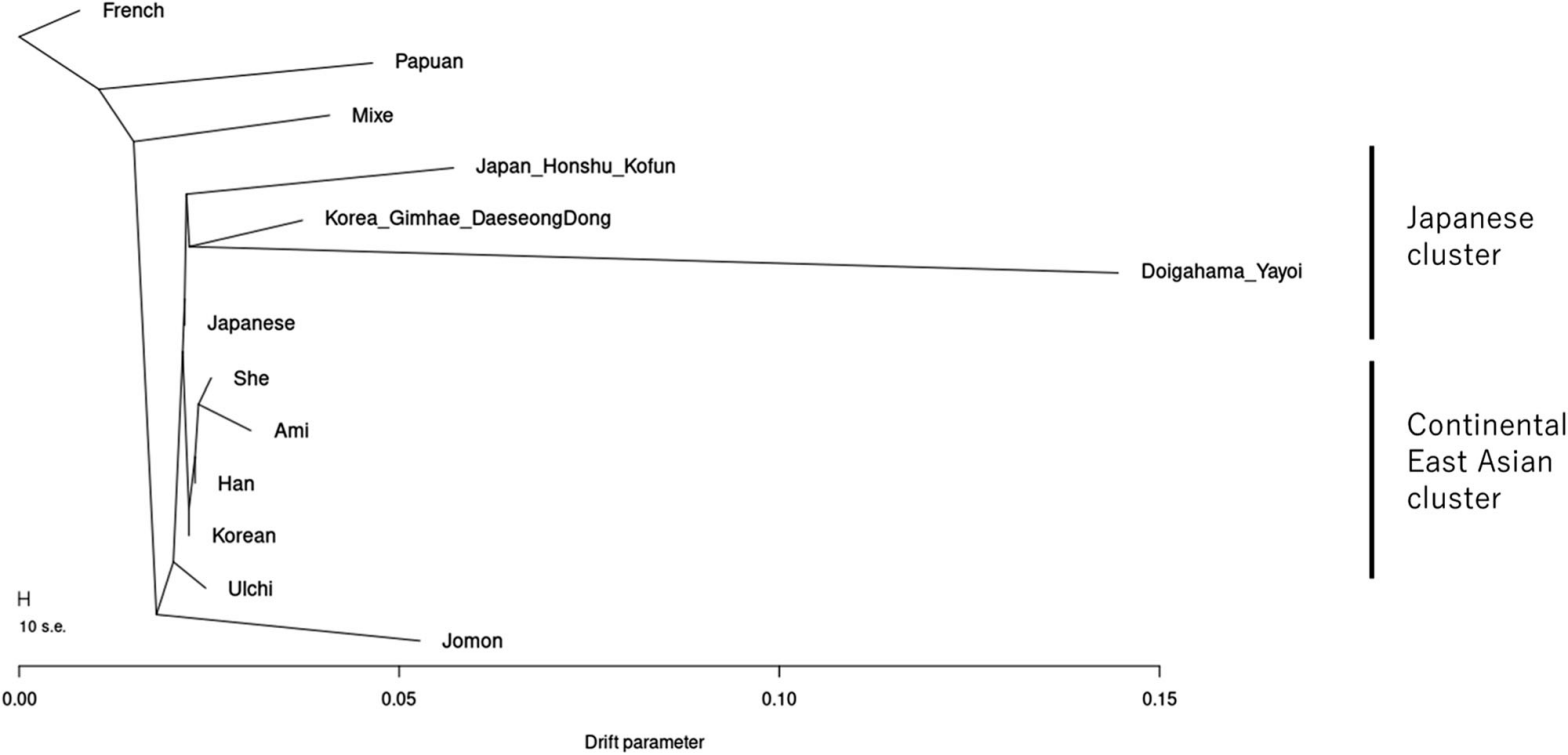
Phylogenetic tree reconstructed using Treemix v1.3. Maximum likelihood phylogenetic tree with European French as the root. Two distinct East Asian clusters, the Japanese cluster, and the continental East Asian cluster, are indicated in the Figure

The TreeMix program [62] was used to explore the phylogenetic relationship of the Doigahama Yayoi individual with East Asian populations (Fig. 4). A tree assumed admixture events was not presented in this study, as none of the plausible scenarios were reproduced (data not shown). In the TreeMix tree, the Jomon population diverged from the other East Asian populations before the divergence of the continental East Asian cluster and the Japanese cluster, supporting that the Jomon lineage is phylogenetically located basal to other East Asian populations.

East Asian populations were divided into two distinct clusters in Fig. 4. One cluster included continental East Asian populations, such as modern Korean, Han Chinese, She, and Ami (referred to as the continental East Asian cluster). The other cluster comprised modern Japanese, the Doigahama Yayoi individual, Japanese_Honshu_Kofun, and Korea_Gimhae_DaeseongDong populations (referred to as the Japanese cluster). Among East Asian populations, the modern Korean population was identified as the closest to the common ancestor of the Japanese cluster. This observation is consistent with the results from PCA (Fig. 2). These results indicate that Korean individuals are genetically closer to Japanese than Han Chinese are.

The ancient Korean population, Korea_Gimhae_DaeseongDong, was included in the Japanese cluster (Fig. 4). This apparent clustering may be due to gene flow from the Jomon population into the Korea_Gimhae_DaeseongDong population, as suggested in [56]. The relationship between the Korea_Gimhae_DaeseongDong population and Japanese populations, as inferred from the TreeMix tree, should be carefully examined in future studies.

### Populations genetically close to the Doigahama Yayoi individual

**Fig. 5:**
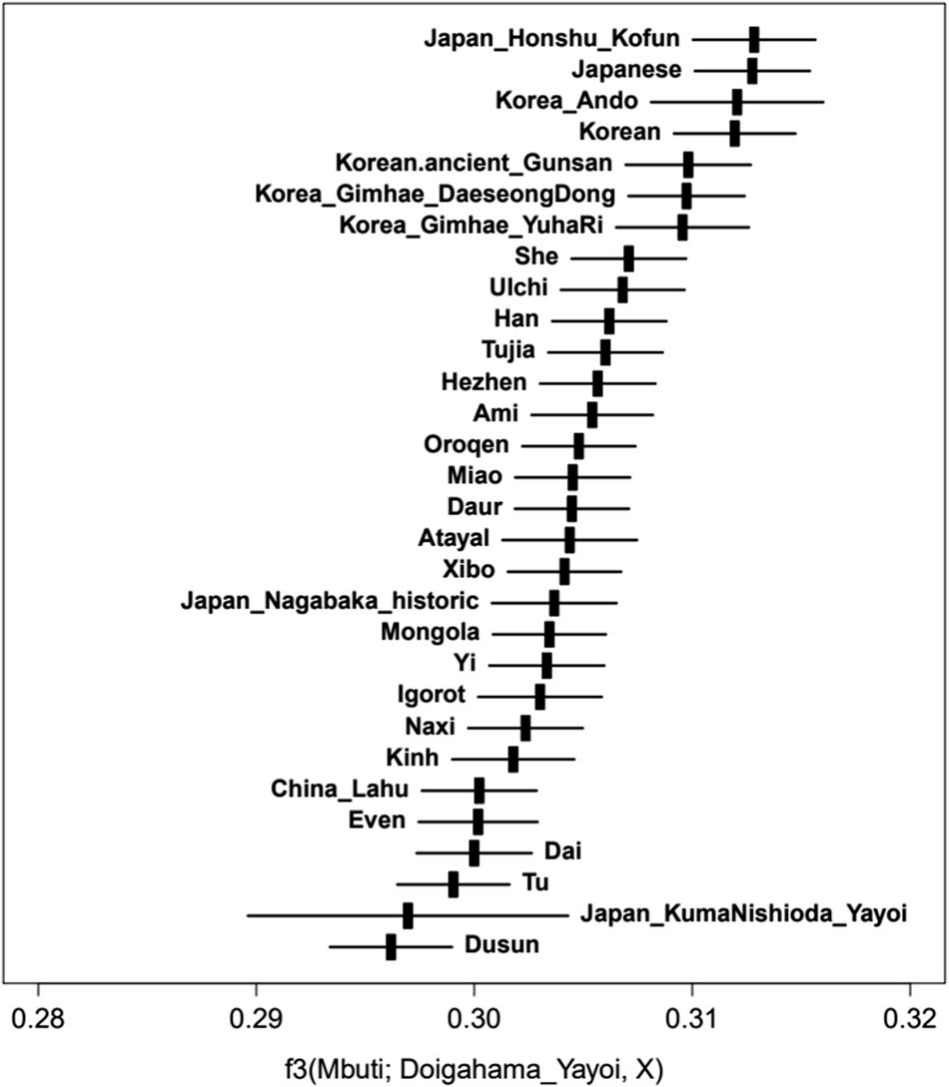
Outgroup *f*3 result illustrating the shared genetic drift with Doigahama Yayoi. The top 30 populations with the highest outgroup *f*3-statistics in our dataset. The error bars represent the range of |*Z* | ≤ 1

The analysis of outgroup *f*3-statistic, a measure revealing shared drift between two populations [63], revealed that Japanese populations (ancient Japan_Honshu_Kofun and modern Japanese) were genetically the closest to the Doigahama Yayoi individual, with ancient and modern Korean populations following closely (Fig. 5). Among ancient Koreans, Korean_Ando exhibited the closest affinity with the Doigahama Yayoi individual, suggesting that immigrants to the Japanese Archipelago during the Yayoi period were derived from populations genetically closer to Korean_Ando than to ancient Koreans from Gunsan and Gimhae. The *f*3-statistics for ancient and modern Japanese, and ancient and modern Korean populations were larger than those for the other modern Asian populations, although their error bars, representing the range of |*Z* | ≤ 1, overlapped with those of some Asian populations.

Additionally, *f*4(Mbuti, Doigahama_Yayoi; modern Japanese or modern Korean, X) was calculated to confirm whether the Japanese and Korean populations are the closest modern populations to the Doigahama Yayoi individual. Populations X were categorized based on the boundary of |*Z* | = 3. While no populations X with *Z* ≥ 3 were observed, populations X showing −3 < *Z* < 3 were detected only when ancient Japanese, modern Japanese, ancient Korean, or modern Korean population was X (Table S5). In all the other models, the *Z* values were under −3. Therefore, at least among the modern populations tested in this study, it was concluded that there are no populations genetically close to the Doigahama individual to the extent comparable to the Japanese and Korean populations.

### The genetic affinity between the Doigahama Yayoi individual and Jomon individuals

The *f*4 test in the form of *f*4(Mbuti, Doigahama_Yayoi; Jomon1, Jomon2) was performed to identify the Jomon population with the highest genetic affinity to the Doigahama Yayoi individual (Figure S3). In essence, the *f*4-statistic becomes positive when the Doigahama Yayoi individual is genetically closer to Jomon2 than to Jomon1, and conversely, becomes negative. This analysis included Jomon populations from various periods and sites. Given the limited number of ancient genomes in each population, the results were interpreted using a lenient criterion of |*Z* | = 2. The *f*4(Mbuti, Doigahama_Yayoi; Japan_Shikoku_LateJomon, the other Jomon individuals) produced negative values with *Z* < −2, indicating that Japan_Shikoku_LateJomon exhibited a significantly stronger affinity to the Doigahama Yayoi individual compared to the other Jomon individuals.

Notably, the Shikoku region, where the Japan_Shikoku_LateJomon was excavated, is geographically closest to the Doigahama site among the regions where the other Jomon samples in the present dataset were excavated (Figure S1). This observation implies that the Jomon-related ancestry of the Doigahama Yayoi individual was derived from Jomon people who lived in areas close to Yamaguchi Prefecture. Although decisive evidence has not yet been reported due to the limited number of high-quality ancient genomes, the present result hints at possible population differentiation among the Jomon population. This should be further investigated using high-quality Jomon genomes from various sites across the Japanese Archipelago.

### Admixture modeling of Doigahama Yayoi Individual

The *f*4 ratio test (Fig. 1) was conducted to assess the model fit and estimate the proportion of Jomon-related ancestry in ancient and modern Japanese individuals including the Doigahama Yayoi individual. Admixture proportions falling outside the [0, 1] interval indicate a lack of fit in the model. In Table S6, admixture proportions for such models are marked in red. The model aligned well when modern Korean was assigned as “b” and the other East Asian as “a” in Fig. 1. However, the reverse scenario assuming the other East Asian as “b” and modern Korean as “a” did not show a good fit, as the admixture proportion (alpha) exceeded 1. Either model assuming Hezhen or Oroqen as “b”, the populations genetically close to Northeastern Siberian, did not fit well. This appears to be due to the shared ancestry between Northeastern Siberian and Jomon lineages [23, 24].

**Fig. 6:**
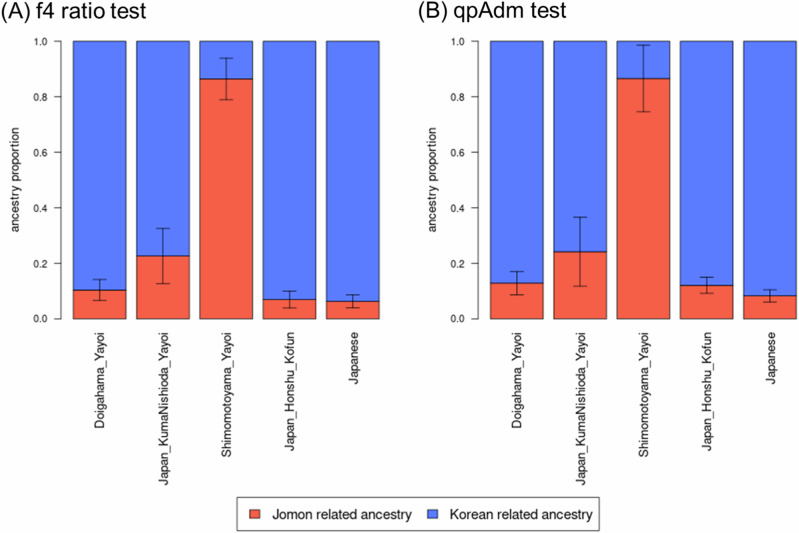
Admixture proportions estimated for two-way admixture model. **A** Admixture proportions estimated in the *f*4 ratio test. **B** Admixture proportions estimated in the qpAdm test. The error bars represent the range of |*Z* | ≤ 1

Therefore, admixture proportions were estimated for the Japanese populations, assuming Jomon and modern Korean as the sources of admixture and designating French and Han as “o” and “a”, respectively (Fig. 6A and Table S6). In this model, the Doigahama Yayoi individual was estimated to have 89.6% of Korean-related ancestry and 10.4% of Jomon-related ancestry. The Kofun individuals and modern Japanese also showed approximately 6–7% of Jomon-related ancestry. On the other hand, the proportion of Jomon-related ancestry of the Shimomotoyama Yayoi individual was notably higher than the others. This is consistent with the result from the PCA, in which the Simomotoyama Yayoi individuals were located close to Jomon people (Fig. 2B).

Finally, we performed a qpAdm analysis to test the admixture model for the Japanese populations, including the Doigahama Yayoi individual (Tables S7 and S8). In this analysis, models with very low *P*-values are incompatible with the data and can be rejected, and models with inferred admixture proportions outside the [0, 1] interval are nonsensical. Therefore, such models were considered unfit for the data. As for the two-way admixture model (Table S7), models assuming Jomon and Han ancestries did not fit for the Doigahama Yayoi individual. In contrast, models assuming Jomon and Korean ancestries provided a better fit not only for the Doigahama Yayoi individual but also for the Kofun individuals and modern Japanese. The bar plots of admixture proportions for the Japanese populations obtained from the qpAdm analysis are shown in Fig. 6B.

In the qpAdm test, the Doigahama Yayoi individual was explained as having 12.9% Jomon-related ancestry and 87.1% Korean-related ancestry (Fig. 6B and Table S7). Although qpAdm analysis is notably sensitive to the choice of the outgroup population set, it yielded similar results with the *f*4 ratio test mentioned above, indicating that the outgroup set in our qpAdm analysis was appropriate.

Using the same outgroup set, we also examined the suitability of three-way admixture models assuming three sources of ancestry: Jomon-related, East Asian-related, and Northeast Asian-related ancestries. In this analysis, Han or Modern Korean individuals were used as the source for East Asian-related ancestry, while the China-HMMH-MN individual was used as the source for Northeast Asian-related ancestry (Table S8). The China-HMMH-MN individual is an ancient sample excavated from the Haminmangha site in Kezuozhongqi, Inner Mongolia, China. We selected the China-HMMH-MN individual as the source for Northeast Asian-related ancestry because it was used for this purpose in a previous study [25] that proposed a three-way admixture model. When Han was regarded as the East Asian population, none of the three-way admixture models produced appropriate admixture proportions for the Japanese populations (i.e., one or more inferred admixture proportions fell outside the [0, 1] range). When modern Korean was used as a source for East Asian-related ancestry, the admixture proportions were within the acceptable range for the Doigahama Yayoi individual and the Japan_Honshu_Kofun. However, the admixture proportions of China_HMMH_MN were smaller than their standard errors in the cases where the Doigahama Yayoi individual and the Japan_Honshu_Kofun were the targets, indicating that the estimated admixture proportions were not strongly supported. Thus, our analysis did not yield any positive results supporting the conclusion that the three-way admixture model is superior to the two-way admixture model, although the three-way admixture models examined were limited.

## Discussion

One of the important findings of this study is that, in all analyses, among modern populations, the Korean population exhibited more genetic similarity to the Doigahama Yayoi individual than any other East Asian populations, except for the Japanese. This suggests that immigrants to the Japanese Archipelago during the Yayoi period primarily originated from the Korean Peninsula. Thus, genetic studies on the admixture of Jomon people and immigrants should first consider the possibility that Koreans were the major source of immigrants to the Japanese Archipelago. If the most plausible source population is not used in admixture modeling, the results can significantly deviate from true history.

In this study, modern and ancient Korean individuals were found to possess substantial Northeastern Eurasian-related and East Asian-related components, consistent with previous studies [73, 74]. Therefore, the admixture of the Korean population with the Jomon population, followed by genetic drift within the admixed population in the Japanese Archipelago, has the potential to explain the major genetic ancestries observed in the Japanese. It is imperative to emphasize that the presence of both East Asian-related and Northeastern Siberian-related components in Japanese genomes, in addition to the Jomon component, does not necessarily imply that two separate populations — one with Northeastern Siberian-related ancestry during the Yayoi period and another with East Asian-related ancestry during the Kofun period — migrated independently to the Japanese Archipelago.

A previous study claimed that the Northeastern Eurasian-related component found in both the Shimomotoyama Yayoi and Kofun individuals was introduced to the Japanese Archipelago during the Yayoi period, and a new East Asian-related component observed in Kofun individuals but not in Yayoi individuals emerged during the Kofun period [25]. However, we found that the Doigahama Yayoi individual dated older than the Shimomotoyama Yayoi individuals had not only Jomon and Northeastern Siberian-related ancestries but also a substantial East Asian-related ancestry (Fig. 3B). The present analysis of admixture modeling for Yayoi, Kofun, and modern Japanese individuals strongly supported a two-way admixture model assuming Jomon-related and Korean-related ancestries (Table S7), although a three-way admixture model was proposed in a previous study [25]. The discrepancy between the two studies may have arisen from the unique genetic profile of the Shimomotoyama Yayoi individuals analyzed in the previous study [25]. These Shimomotoyama Yayoi individuals have been reported to be genetically close to Jomon people [26]. Consistently, in this study, the Shimomotoyama Yayoi individuals were positioned close to Jomon people in the PCA plot (Fig. 2B), and the estimated proportion of Jomon-related ancestry in the Shimomotoyama Yayoi individuals was much higher than that in the Doigahama Yayoi individual (Fig. 3B). Since the immigrants came from continental East Asia, Yayoi individuals who are genetically more distant from Jomon people and closer to continental East Asians seem to be more suitable for the analysis to clarify the origins of immigrants. In this context, the Doigahama Yayoi individual, rather than the Shimomotoyama Yayoi individuals, should be used, as the representative of Yayoi individual, in the study of admixture in the Japanese Archipelago.

The analysis of outgroup *f*3 indicated that the Doigahama Yayoi individual shared more genetic drift with modern Korean and Korean_Ando individuals than with the other ancient Korean individuals (Fig. 5). Thus, the immigrants to the Japanese Archipelago during the Yayoi period may have been derived from populations genetically closer to modern Korean or Korean_Ando. At present, it is not clear from which part of the Korean Peninsula the immigrants originated; analyzing many ancient Korean genomes from around 3,000 years ago, when the Yayoi period started, may help determine the main regions from which the immigrants came on the Korean Peninsula.

East Eurasian populations have been reported to exhibit a genetic cline along a North-South axis [75, 76]. It is expected that in continental East Eurasia, more northerly populations possess a higher proportion of Northeastern Siberian-related ancestry, while more southerly populations tend to have more East Asian-related ancestry. Considering all the results from the present study, the most plausible scenario is as follows (Figure S4): The Korean population, possessing both East Asian-related and Northeastern Siberian-related ancestries, migrated continuously to the Japanese Archipelago from the Yayoi period to the Kofun period. This migration involved admixture with the local Jomon people. Modern Mainland Japanese are considered descendants of this admixed population. Future analysis of the genomes of Yayoi and Kofun individuals from various sites of the Japanese Archipelago would shed light on how the immigrants spread across the Japanese Archipelago while admixing with the Jomon people.

## Supplementary information

Supplementary information
