# Supplementary material for: Genetic analysis of a Yayoi individual from the Doigahama site provides insights into the origins of immigrants to the Japanese Archipelago
Source: J Hum Genet. 2024 Oct 15;70(1):47–57. doi: 10.1038/s10038-024-01295-w (PMC11700843; doi:10.1038/s10038-024-01295-w)
Supplement: Supplementary file 1 — Supplementary information [file 10038_2024_1295_MOESM1_ESM.docx]

**Supplementary information**

**Genetic Analysis of a Yayoi Individual from the Doigahama Site Provides Insights into the Origins of Immigrants to the Japanese Archipelago**

Jonghyun Kim^1^, Fuzuki Mizuno^2, *^, Takayuki Matsushita^3^, Masami Matsushita^3^, Saki Aoto^4^, Koji Ishiya^1^, Mami Kamio^1^, Izumi Naka^1^, Michiko Hayashi^2^, Kunihiko Kurosaki^2^, Shintaroh Ueda^1, 2^, Jun Ohashi^1, *^

1. Department of Biological Sciences, Graduate School of Science, The University of Tokyo, Tokyo, 113-0033, Japan.

2. Department of Legal Medicine, Toho University School of Medicine, Tokyo, 143-8540, Japan.

3. The Doigahama Site Anthropological Museum, Yamaguchi, 759-6121, Japan

4. Medical Genome Center, National Research Institute for Child Health and Development, Tokyo, 157-8535, Japan.

^*^To whom correspondence may be addressed:

Fuzuki Mizuno, Ph.D.

Department of Legal Medicine, Toho University School of Medicine, 5-21-16, Omori-Nishi, Ota-ku, Tokyo, 143-8540, Japan.

Email: fuzuki.mizuno@med.toho-u.ac.jp

Jun Ohashi, Ph.D.

Department of Biological Sciences, Graduate School of Science, The University of Tokyo, 7-3-1, Hongo, Bunkyo-ku, Tokyo, 113-0033, Japan.

Email: jun_ohashi@bs.s.u-tokyo.ac.jp

**Table S1.** A list of modern populations used in this study.

| **Regions** | **Populations (n=sample number)** |
| --- | --- |
| Africa | BantuKenya(6), BantuSA(5), BantuSA_Herero(2), BantuSA_Ovambo(1), Biaka(19), Ju_hoan_North(5), Mandenka(17), Mbuti(10), Mozabite(22), Yoruba(21), Dinka(4), Esan(2), Gambian(2), Igbo(2), Khomani_San(2), Kongo(1), Lemande(2), Luhya(2), Luo(2), Masai(2), Mende(2), Saharawi(2), Somali(1) |
|  |  |
| West Eurasia | Adygei(13), Basque(21), BedouinA(25), BedouinB(19), Druze(39), French(25), Italian_North(19), Orcadian(15), Palestinian(35), Russian(24), Sardinian(27), Albanian(1), Armenian(2), Bulgarian(2), Chechen(1), Cretan(2), Czech(1), English(2), Estonian(2), Finnish(2), French(1), Georgian(2), Greek(2), Hungarian(2), Icelandic(2), Iranian(2), Jew_Iraqi(2), Jew_Yemenite(2), Jordanian(3), Lezgin(2), Norwegian(1), Polish(1), Russia_Abkhasian(2), Russia_NorthOssetian(2), Saami(2), Samaritan(1), Spanish(2), Tajik(2), Turkish(2) |
|  |  |
| Central South Asia | Balochi(20), Brahui(22), Burusho(22), Hazara(13), Kalash(21), Makrani(20), Pathan(17), Sindhi_Pakistan(14), Uyghur(10), Bengali(2), Brahmin(2), Irula(2), Kapu(2), Khonda_Dora(1), Kusunda(2), Madiga(2), Mala(2), Punjabi(3), Relli(2), Yadava(2) |
|  |  |
| Central Asia, Siberia | Yakut(20), Aleut(2), Altaian(1), Chukchi(1), Eskimo_Chaplin(1), Eskimo_Naukan(2), Eskimo_Sireniki(2), Even(2), Itelmen(1), Kyrgyz_Kyrgyzstan(2), Mansi(2), Tubalar(2), Ulchi(2) |
|  |  |
| East Asia | Cambodian(7), China_Lahu(7), Dai(9), Daur(8), Han(42), Hezhen(8), Japanese(28), Miao(10), Mongola(6), Naxi(7), Oroqen(9), She(10), Tu(10), Tujia(9), Xibo(7), Yi(10), Ami(2), Atayal(1), Burmese(2), Kinh(2), Korean(2), Naxi(1) |
|  |  |
| America | Karitiana(10), Mayan(18), Piapoco(6), Pima(11), Surui(5), Chane(1), Chipewyan(2), Cree(2), Mexico_Zapotec(2), Mixe(3), Mixtec(2), Nahua(1), Quechua(2) |
|  |  |
| Oceania | Nasioi(10), Papuan(14), Australian(2), Dusun(2), Hawaiian(1), Igorot(2), Maori(1) |
|  |  |

**Table S2.** Information of ancient individuals used in this study.

| **Region (period)** | **Population name** | **Sample ID** |
| --- | --- | --- |
| Mainland of Japan (Jomon) | Japan_Shikoku_InitialJomon | JpKa6904 |
|  | Japan_Honshu_EarlyJomon_Funagara | JpFu1 |
|  | Japan_Honshu_EarlyJomon_Odake | JpOd274 |
|  |  | JpOd6 |
|  |  | JpOd181 |
|  |  | JpOd282 |
|  | Japan_Honshu_MidLateJomon | JpKo2 |
|  |  | JpKo13 |
|  | Japan_Shikoku_LateJomon | JpHi01 |
|  | Rokutsu_Jomon | I13882 |
|  |  | I13883 |
|  |  | I13884 |
|  |  | I13885 |
|  |  | I13886 |
|  | Funadomari_Jomon | Fun23 |
|  |  | Fun5 |
|  | Ikawazu_Jomon | IK002 |
| Mainland of Japan (Yayoi) | Doigahama_Yayoi | D1604 |
|  | Japan_KumaNishioda_Yayoi | YAK002 |
|  | Shimomotoyama_Yayoi | Shimomotoyama2 |
|  |  | Shimomotoyama3 |
| Mainland of Japan (Kofun) | Japan_Honshu_Kofun | JpIw31 |
|  |  | JpIw32 |
|  |  | JpIw33 |
| Ancient Ryukyu | Nagabaka_4000BP | NAG016 |
|  | Nagabaka_2800BP | NAG012 |
|  |  | NAG019 |
|  |  | NAG037 |
|  |  | NAG038 |
|  | Nagabaka_historic | NAG007 |
|  |  | NAG035 |
|  |  | NAG036 |
|  |  | NAG039 |
| Korea | Korea_Yondaedo | TYD006 |
|  |  | TYD007 |
|  | Korea_Ando | AND001 |
|  |  | AND004 |
|  | Korea_Changhang | GDI002 |
|  |  | GDI008 |
|  | Korea_Yokchido | TYJ001 |
|  | Korea_Taejungni | DAJ001 |
|  | Korea_Gimhae_DaeseongDong | AKG_3421 |
|  |  | AKG_10203 |
|  |  | AKG_10204 |
|  |  | AKG_10207 |
|  |  | AKG_10209 |
|  |  | AKG_10210 |
|  |  | AKG_10218 |
|  | Korea_Gimhae_YuhaRi | AKG_3420 |
|  | Korean.ancient_Gunsan | GUC001 |
|  |  | GUC002 |
|  |  | GUC003 |
|  |  | GUC004 |
|  |  | GUC005 |
|  |  | GUC007 |

**Table S3.** Number of mapped reads to sex chromosomes.

| **ID** | **X-chromosome** | **Y-chromosome** | ***R*_Y_ index** | **95% CI** |
| --- | --- | --- | --- | --- |
| D1604 | 5,309,287 | 2,695 | 0.00051 | 0.00049-0.00053 |

**Table S4.** Summary of haploid genome calling using pileupcaller.

| **ID** | **Total Target Sites** | **Non Missing Calls** | **mean coverage of target sites** |
| --- | --- | --- | --- |
| D1604 | 1150639 | 971776 | 2.085 x |

**Table S5.** The models in which *Z* value of *f*4(Mbuti, Doigahama_Yayoi; Japanese or Korean, X) > -3.

| **Target** | **X** | ***f*4** | ***Z*** | **nBABA** | **nABBA** | **nSNPs** |
| --- | --- | --- | --- | --- | --- | --- |
| Modern Korean | Modern Japanese | 0.000187 | 0.663 | 48874 | 48697 | 941715 |
| Modern Korean | Japan_Honshu_Kofun | 0.000181 | 0.514 | 48917 | 48747 | 940838 |
| Modern Korean | Japan_KumaNishioda_Yayoi | -0.001331 | -0.965 | 1161 | 1191 | 22911 |
| Modern Korean | Korea_Ando | -0.000069 | -0.096 | 6225 | 6233 | 119841 |
| Modern Korean | Korea_Gimhae_DaeseongDong | -0.000569 | -1.747 | 49098 | 49633 | 941664 |
| Modern Korean | Korea_Gimhae_YuhaRi | -0.000518 | -1.079 | 46475 | 46940 | 898088 |
| Modern Korean | Korean.ancient_Gunsan | -0.000282 | -0.707 | 40952 | 41177 | 796066 |
| Modern Japanese | Modern Korean | -0.000187 | -0.663 | 48697 | 48874 | 941715 |
| Modern Japanese | Japan_Honshu_Kofun | 0.000019 | 0.075 | 50056 | 50038 | 964163 |
| Modern Japanese | Japan_KumaNishioda_Yayoi | -0.001850 | -1.484 | 1177 | 1220 | 23337 |
| Modern Japanese | Korea_Ando | -0.000765 | -1.286 | 6245 | 6339 | 121926 |
| Modern Japanese | Korea_Gimhae_YuhaRi | -0.000689 | -1.717 | 47692 | 48325 | 919666 |
| Modern Japanese | Korean.ancient_Gunsan | -0.000428 | -1.440 | 41911 | 42258 | 812626 |

**Table S6.** The results of the *f*4 ratio test targeting Doigahama Yayoi.

| **Population** | **a=Modern Korean, b=East Asian** | | | **a=East Asian, b=Modern Korean** | | |
| --- | --- | --- | --- | --- | --- | --- |
|  | alpha | 1-alpha | SE | alpha | 1-alpha | SE |
| Ami | 1.238631 | -0.238631 | 0.072382 | 0.968314 | 0.031686 | 0.076369 |
| Atayal | 1.209995 | -0.209995 | 0.090247 | 0.979587 | 0.020413 | 0.095206 |
| Burmese | 2.726312 | -1.72631 | 0.325266 | 0.859635 | 0.140365 | 0.069661 |
| Cambodian | 2.852454 | -1.85245 | 0.294429 | 0.818890 | 0.18111 | 0.060907 |
| China_Lahu | 1.369675 | -0.369675 | 0.070892 | 0.904541 | 0.095459 | 0.056520 |
| Dai | 1.274940 | -0.27494 | 0.063123 | 0.827373 | 0.172627 | 0.049468 |
| Daur | 1.475406 | -0.475406 | 0.080952 | 0.995592 | 0.004408 | 0.044850 |
| Han | 1.081213 | -0.081213 | 0.046930 | 0.896094 | 0.103906 | 0.037919 |
| Hezhen | 1.379637 | -0.379637 | 0.072743 | 1.020386 | -0.020386 | 0.045709 |
| Kinh | 1.281806 | -0.281806 | 0.077459 | 0.909492 | 0.090508 | 0.060913 |
| Miao | 1.108948 | -0.108948 | 0.052526 | 0.867048 | 0.132952 | 0.043117 |
| Mongola | 1.458288 | -0.458288 | 0.076877 | 0.958762 | 0.041238 | 0.045338 |
| Naxi | 1.241008 | -0.241008 | 0.062607 | 0.882889 | 0.117111 | 0.042464 |
| Oroqen | 1.588440 | -0.58844 | 0.086729 | 1.046531 | -0.046531 | 0.049848 |
| She | 1.092731 | -0.092731 | 0.051736 | 0.954985 | 0.045015 | 0.044605 |
| Thai | 2.102911 | -1.10291 | 0.195080 | 0.831584 | 0.168416 | 0.067221 |
| Tu | 1.716581 | -0.716581 | 0.101780 | 0.903914 | 0.096086 | 0.039318 |
| Tujia | 1.059241 | -0.059241 | 0.046679 | 0.882504 | 0.117496 | 0.040751 |
| Xibo | 1.407177 | -0.407177 | 0.072443 | 0.970784 | 0.029216 | 0.043646 |
| Yi | 1.231090 | -0.23109 | 0.057515 | 0.914960 | 0.08504 | 0.044185 |

**Table S7.** The result of qpAdm admixture modeling analysis for two-way admixture model.

| **Two-way admixture model (1: Jomon, 2: Han)** | | | | | | | |
| --- | --- | --- | --- | --- | --- | --- | --- |
| **Target** | ***P*-value** | **c1** | **c2** | **c3** | **SE1** | **SE2** | **SE3** |
| Doigahama_Yayoi | 0.00385400732 | 0.170 | 0.830 | - | 0.038 | 0.038 | - |
| Japan_KumaNishioda_Yayoi | 0.105096888 | 0.305 | 0.695 | - | 0.110 | 0.110 | - |
| Shimomotoyama_Yayoi | 0.944799334 | 0.853 | 0.147 | - | 0.108 | 0.108 | - |
| Japan_Honshu_Kofun | 2.24898816e-08 | 0.152 | 0.848 | - | 0.023 | 0.023 | - |
| Modern Japanese | 2.48560586e-34 | 0.106 | 0.894 | - | 0.010 | 0.010 | - |
| **Two-way admixture model (1: Jomon, 2: Modern Korean)** | | | | | | | |
| **Target** | ***P*-value** | **c1** | **c2** | **c3** | **SE1** | **SE2** | **SE3** |
| Doigahama_Yayoi | 0.842802462 | 0.129 | 0.871 | - | 0.042 | 0.042 | - |
| Japan_KumaNishioda_Yayoi | 0.281292151 | 0.242 | 0.758 | - | 0.124 | 0.124 | - |
| Shimomotoyama_Yayoi | 0.905349491 | 0.866 | 0.134 | - | 0.120 | 0.120 | - |
| Japan_Honshu_Kofun | 0.159743651 | 0.121 | 0.879 | - | 0.029 | 0.029 | - |
| Modern Japanese | 0.0907712115 | 0.083 | 0.917 | - | 0.022 | 0.022 | - |

The notation of c*i* (*i*=1 and 2) respectively indicates the admixture proportion from the *i*-th ancestry, and SE*i* indicates the standard error of c*i*.

**Table S8.** The result of qpAdm admixture modeling analysis for three-way admixture model.

| **Three-way admixture model (1: Jomon, 2: Han, and 3: China_HMMH_MN)** | | | | | | | |
| --- | --- | --- | --- | --- | --- | --- | --- |
| **Target** | ***P*-value** | **c1** | **c2** | **c3** | **SE1** | **SE2** | **SE3** |
| Doigahama_Yayoi | 0.676729272 | -0.006 | 0.595 | 0.411 | 0.074 | 0.093 | 0.142 |
| Japan_KumaNishioda_Yayoi | 0.363670302 | -1.463 | -0.686 | 3.149 | 7.928 | 5.197 | 13.086 |
| Shimomotoyama_Yayoi | 0.996733416 | 1.094 | 0.228 | -0.323 | 0.238 | 0.166 | 0.255 |
| Japan_Honshu_Kofun | 0.340903025 | -0.002 | 0.586 | 0.416 | 0.050 | 0.066 | 0.100 |
| Modern Japanese | 0.389963923 | -0.016 | 0.682 | 0.334 | 0.025 | 0.035 | 0.051 |
| **Three-way admixture model (1: Jomon, 2: Modern Korean, and 3: China_HMMH_MN)** | | | | | | | |
| **Target** | ***P*-value** | **c1** | **c2** | **c3** | **SE1** | **SE2** | **SE3** |
| Doigahama_Yayoi | 0.940134142 | 0.083 | 0.833 | 0.084 | 0.081 | 0.124 | 0.181 |
| Japan_KumaNishioda_Yayoi | 0.643120491 | 2.294 | 2.184 | -3.478 | 7.712 | 6.259 | 13.930 |
| Shimomotoyama_Yayoi | 0.99260954 | 1.154 | 0.231 | -0.386 | 0.246 | 0.186 | 0.299 |
| Japan_Honshu_Kofun | 0.133146288 | 0.083 | 0.782 | 0.135 | 0.073 | 0.117 | 0.179 |
| Modern Japanese | 0.0970374294 | 0.100 | 0.948 | -0.048 | 0.063 | 0.110 | 0.166 |

The notation of c*i* (*i*=1, 2, and 3) respectively indicates the admixture proportion from the *i*-th ancestry, and SE*i* indicates the standard error of c*i*.


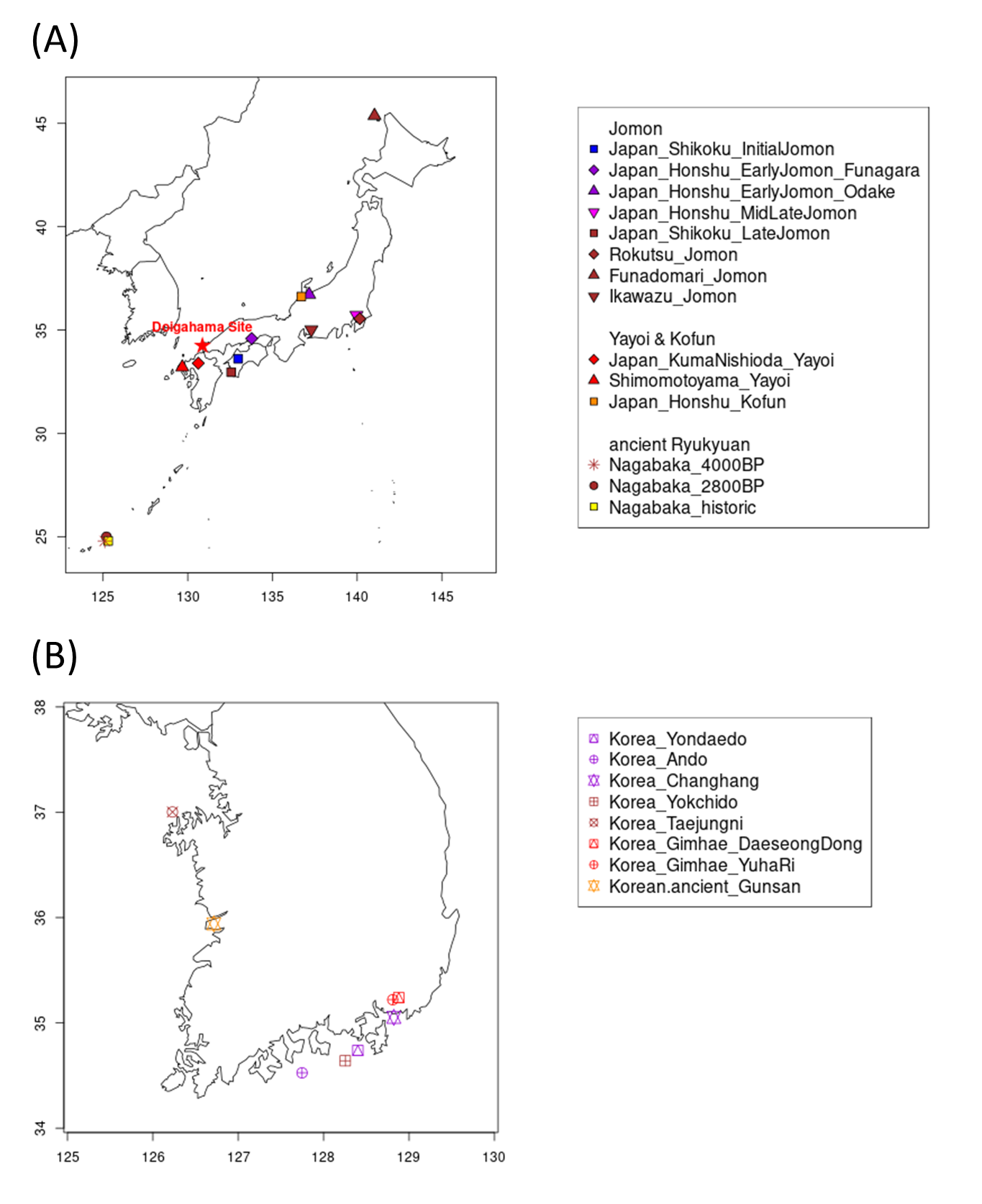


**Figure S1.** The location of Doigahama site.

We illustrated the location of the Doigahama site and other main archaeological sites where the ancient samples in Table S2 were excavated. The individuals are color-coded as follows: the Initial Jomon period (8500-5000 BC) in blue, the Early Jomon period (5000-3520 BC) in purple, the Middle Jomon period (3520-2470 BC) in pink, the Late-Final Jomon period (2470-500 BC) in brown, Yayoi period (500 BC-300 AC) in red, the Kofun period (300-538 AC) in orange, and Edo period (1603-1868 AC) in yellow.

(A) archaeological sites in mainland Japan and Ryukyu (B) archaeological sites in South Korea


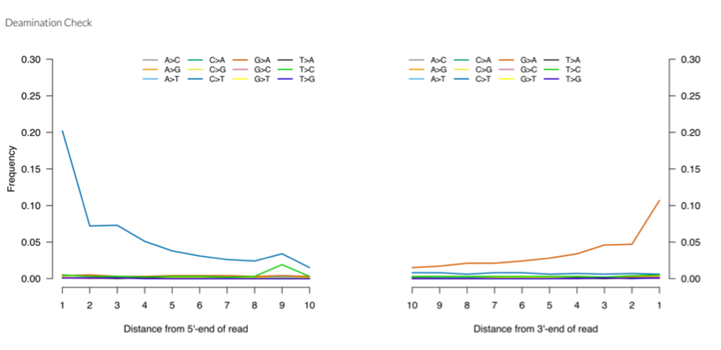


**Figure S2.** The deamination pattern of the double stranded library of D1604.

The mismatch frequency is relative to the reference as a function of read position; C to T in blue and G to A in red.


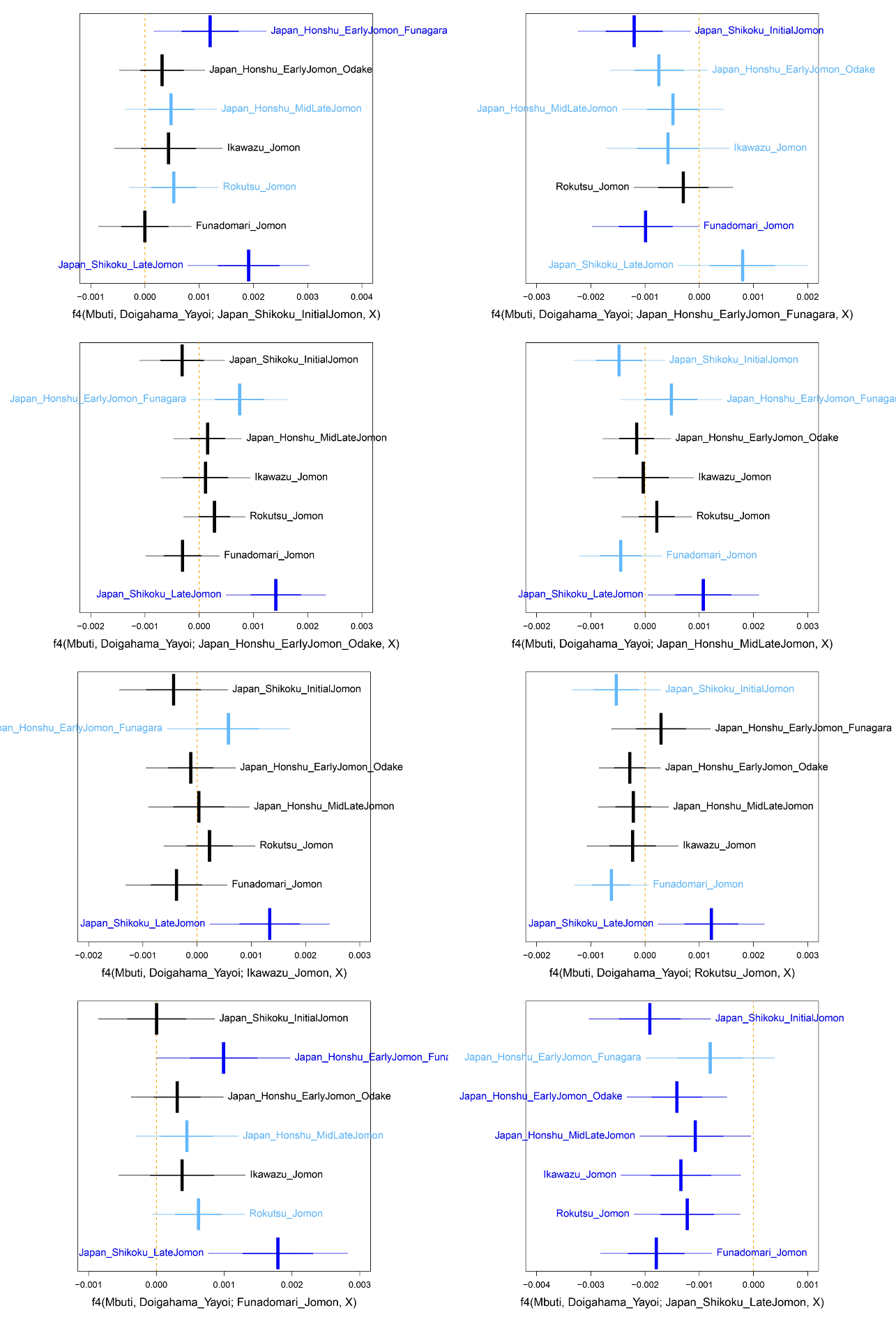


**Figure S3.** *f*4(Mbuti, Doigahama_Yayoi; Jomon1, Jomon2).

The thick error bars represent the range of |*Z*|≤1, and the thin error bars represent the range of |*Z*|≤2. The cases with |*Z*|≤1, 1< |*Z*|< 2, and |*Z*|≥2 are presented in black, sky blue, and deep blue, respectively.


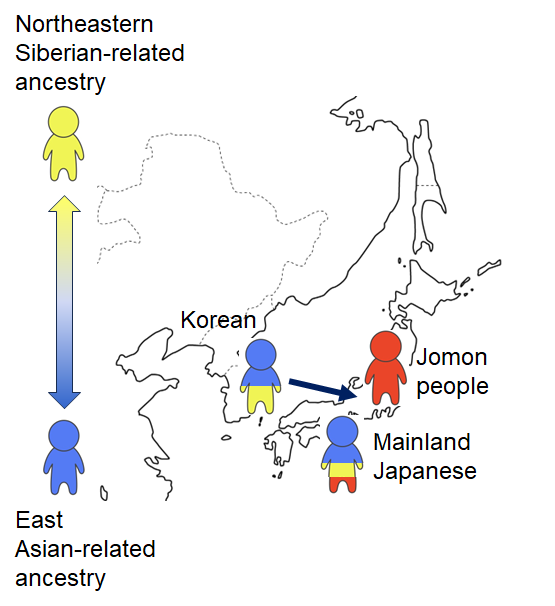


**Figure S4.** Two-way admixture model proposed in this study.

The Korean population, possessing both East Asian-related and Northeastern Siberian-related ancestries, migrated from the Korean Peninsula to the Japanese Archipelago, where they admixed with Jomon people. This pattern of migration and admixture continued from the Yayoi to the Kofun period. Modern Mainland Japanese are the descendants of this admixed population.

**REFERENCE**

1. Cooke NP, Mattiangeli V, Cassidy LM, Okazaki K, Stokes CA, Onbe S, et al. Ancient genomics reveals tripartite origins of Japanese populations. Science advances. 2021;7(38):eabh2419.
2. Gakuhari T, Nakagome S, Rasmussen S, Allentoft ME, Sato T, Korneliussen T, et al. Ancient Jomon genome sequence analysis sheds light on migration patterns of early East Asian populations. Communications biology. 2020;3(1):437.
3. Gelabert P, Blazyte A, Chang Y, Fernandes DM, Jeon S, Hong JG, et al. Northeastern Asian and Jomon-related genetic structure in the Three Kingdoms period of Gimhae, Korea. Current Biology. 2022;32(15):3232-3244.
4. Kanzawa-Kiriyama H, Jinam TA, Kawai Y, Sato T, Hosomichi K, Tajima A, et al. Late Jomon male and female genome sequences from the Funadomari site in Hokkaido, Japan. Anthropological Science. 2019;127(2):83-108.
5. Lee DN, Jeon CL, Kang J, Burri M, Krause J, Woo EJ, Jeong C. Genomic detection of a secondary family burial in a single jar coffin in early Medieval Korea. American Journal of Biological Anthropology. 2022;179(4):585-597.
6. Robbeets M, Bouckaert R, Conte M, Savelyev A, Li T, An DI, et al. Triangulation supports agricultural spread of the Transeurasian languages. Nature. 2021;599(7886):616-621.
7. Shinoda KI, Kanzawa-Kiriyama H, Kakuda T, Adachi N. Genetic characteristics of Yayoi people in northwestern Kyushu: ancient genome analysis of human bones excavated from Shimomotoyama rock shelter, Sasebo, Nagasaki prefecture, Japan. Anthropological Science (Japanese Series). 2019;127:25-43.
8. Wang CC, Yeh HY, Popov AN, Zhang HQ, Matsumura H, Sirak K, et al. Genomic insights into the formation of human populations in East Asia. Nature. 2021;591(7850):413-419.
